# Constrained Sintering in Fabrication of Solid Oxide Fuel Cells

**DOI:** 10.3390/ma9080675

**Published:** 2016-08-09

**Authors:** Hae-Weon Lee, Mansoo Park, Jongsup Hong, Hyoungchul Kim, Kyung Joong Yoon, Ji-Won Son, Jong-Ho Lee, Byung-Kook Kim

**Affiliations:** High-Temperature Energy Materials Research Center, Korea Institute of Science and Technology, Seoul 136-791, Korea; mansoo@kist.re.kr (M.P.); jhong@kist.re.kr (J.H.); hyoungchul@kist.re.kr (H.K.); kjyoon@kist.re.kr (K.J.Y.); jwson@kist.re.kr (J.-W.S.); jongho@kist.re.kr (J.-H.L.); bkkim@kist.re.kr (B.-K.K.)

**Keywords:** solid oxide fuel cell, constrained sintering, electrolyte, cathode, composite

## Abstract

Solid oxide fuel cells (SOFCs) are inevitably affected by the tensile stress field imposed by the rigid substrate during constrained sintering, which strongly affects microstructural evolution and flaw generation in the fabrication process and subsequent operation. In the case of sintering a composite cathode, one component acts as a continuous matrix phase while the other acts as a dispersed phase depending upon the initial composition and packing structure. The clustering of dispersed particles in the matrix has significant effects on the final microstructure, and strong rigidity of the clusters covering the entire cathode volume is desirable to obtain stable pore structure. The local constraints developed around the dispersed particles and their clusters effectively suppress generation of major process flaws, and microstructural features such as triple phase boundary and porosity could be readily controlled by adjusting the content and size of the dispersed particles. However, in the fabrication of the dense electrolyte layer via the chemical solution deposition route using slow-sintering nanoparticles dispersed in a sol matrix, the rigidity of the cluster should be minimized for the fine matrix to continuously densify, and special care should be taken in selecting the size of the dispersed particles to optimize the thermodynamic stability criteria of the grain size and film thickness. The principles of constrained sintering presented in this paper could be used as basic guidelines for realizing the ideal microstructure of SOFCs.

## 1. Introduction

Solid oxide fuel cells and stacks may be considered as an assembly of a number of multicomponent composites which are able to provide multiple functions required for high performance and durability [1,2,3,4]. All of the cell components are integrated in the form of a layered composite that requires strong interfaces without chemical reactions or major process flaws [5,6,7,8,9]. To produce a high-performance solid oxide fuel cell, it is necessary to achieve microstructural uniformity of all of the component layers in order to improve its durability and performance. The electrolyte layers need to be sintered to a nearly full density for gas tightness without abnormal grain growth, while the electrode layers must be prepared with appropriate pore structure and phase connectivity of all constituents for efficient transport of gas, ions, and electrons, and highly active electrode reactions [1,5,8,10,11,12,13,14,15,16,17]. Although ideal microstructures exist for all of the component layers in a solid oxide fuel cell, it is very difficult to obtain the intended microstructures in the presence of mutual interactions between the constituent layers during fabrication process. These interactions produce substantial deviations from the ideal microstructure, whether the cells are developed through the co-firing of multilayered laminates or the post-firing of thick films formed on rigid substrates. In both cases, the individual constituent layers experience substantially different shrinkage rates, resulting in a variety of microstructural heterogeneities and the consequent performance degradation of solid oxide fuel cells at the level of both individual cells and stacks. Therefore, it is important to understand the effect of constrained sintering on microstructure evolution in both multiphase composite components themselves, as well as in cells, particularly from the viewpoint of flaw generation during fabrication and the subsequent structural damages and/or failure during operation [6,7,18,19,20,21,22,23,24]. In SOFC development, it has been acknowledged that constrained sintering generally leads to inadequate film density and unfavorable pore structures [25] and, to address this issue, research efforts have been devoted to understanding the microstructure evolution, stress development and defect formation based on experimental and theoretical approaches [26,27,28,29,30]. For instance, the effects of particle size, size distribution, and sintering temperature on the sintering kinetics and microstructural evolution of a composite electrode were studied based on a three-dimensional Monte Carlo model [30], and the sintering model predicted that constrained sintering leads to an anisotropic microstructure, which becomes more isotropic with further densification [28]. The relationship between stress and the sintering profile has been studied using a continuum model [27], and the stress analysis revealed that the excessive residual stress that develops in constrained sintering is relieved by microcrack formation during subsequent cooling [29]. In the present study, the principles of constrained sintering for fabrication of a porous cathode and a dense electrolyte are presented to further improve our fundamental understanding and provide scientific basis for realizing the ideal architecture of solid oxide fuel cells.

## 2. Constrained Sintering of a Porous Composite Cathode

With respect to electrode performance, an ideal microstructure has been proposed as an interpenetrating phase composite structure with the appropriate porosity and pore-solid phase connectivity, which is expected to provide the maximum triple-phase boundary length, in combination with efficient gas transport [10,11,12,15,16,17]. However, it is difficult to obtain such a composite structure with the appropriate pore structure on a rigid substrate due to the lack of controllability over severely-constrained sintering in the presence of the external constraints imposed by a rigid substrate. This problem has been explicitly illustrated in Barnett’s three-dimensional reconstruction study on a La_1−*x*_Sr*_x_*MnO_3_ (LSM)–yttria-stabilized zirconia (YSZ) composite cathode, in which a considerably high fraction of the triple phase boundary length was inactive due to the discontinuous nature of the three-dimensional network of electrode component phases [17]. In addition, some of the optimum composition studies on La_1−*x*_Sr*_x_*Co*_y_*Fe_1−*y*_O_3_ (LSCF)–gadolinia-doped ceria (GDC) composites by several researchers also revealed that the packing behavior associated with powder characteristics and consolidation methods resulted in an exceptionally wide variation in the optimum compositions in terms of the cathode performance, as shown in Table 1 [11,12,15,16].

The spin coating in [15] suffers from relatively poor packing due to extremely fast drying, which limits particle rearrangement due to the rapid reduction of liquid vehicle. On the other hand, the film prepared in [11] will not have an exceptionally high packing density after slurry coating, but the subsequent CIP (cold isostatic pressing) will force the component particles to rearrange themselves into a high packing density. Therefore, the composite cathode prepared in [11] needed only 36% of GDC to develop a continuous GDC network structure in the LSCF matrix due to the reduced interparticle spacing between the GDC particles by a relatively uniform packing structure with a high packing density. In contrast, the composite cathode prepared in [15] may obtain relatively large interspacing between GDC particles at a similar composition to [11] due to poor packing, requiring further shrinkage of the LSCF matrix to form a continuous GDC network structure. By doing so, the composite cathode should be treated at higher sintering temperature, losing substantial amount of pore volume. Instead, increasing GDC content effectively reduces the interparticle spacing between GDC particles at relatively low packing density, which is beneficial for increasing the phase connectivity of both GDC and LSCF at a given shrinkage. Decreasing shrinkage can assure high porosity of the composite cathode for efficient gas flow.

The particle packing structure controls the critical shrinkage for obtaining connectivity of both the phases and the porosity of the composite cathodes. The optimum compositions determined in [12,16] clearly demonstrated that the initial powder characteristics, particularly the degree of agglomeration, dictate the packing density, microstructure, and resulting performance of the composite cathodes in accordance with the constrained sintering principle. The initial powders prepared in [16] are believed to be less aggregated than those in [12] because the LSCF powder prepared by the glycine nitrate process (GNP) is inherently exposed to a highly-aggregated nature despite its chemical homogeneity. The reduced packing efficiency of the GNP-synthesized LSCF powder will require a substantially higher content of GDC powder in order to obtain relatively small interparticle spacing between GDC particles. The extremely high GDC content, e.g., 65% by volume, in [12], should be attributed to the highly-aggregated nature of the LSCF powder obtained by GNP which is responsible for the poor particle packing density. All of the examples in Table 1 suggest that the packing behavior of individual component powders affects the mixing state, e.g., mixing homogeneity, as well as mixing scale, controlling the optimum composition of the composite cathode by altering the critical shrinkage and microstructure such as phase connectivity and porosity. The mixing scale of the composite plays a predominant role through the packing density in determining the critical shrinkage for the phase connectivity of all participating solid phases.

We, now, investigate the details of the constrained sintering of composite cathodes (on a rigid substrate). When two initial powders were mixed together for composite fabrication, one component usually acts as a continuous matrix phase while the other acts as a dispersed phase depending upon the initial composition and packing efficiency. Therefore, the composite cathode needs to be sintered to a critical matrix shrinkage for the dispersed phase to form a continuous network structure, leading to the maximum triple phase boundary length. In doing so, if the network structure of the dispersed particles is formed at a small shrinkage, the resulting composite cathode is expected to have high porosity. Therefore, it is desirable for the composite cathode to be fabricated with minimum matrix shrinkage as long as it maintains proper interface strength with the electrolyte layer. Thus, the composite cathode should be produced with sufficient interface strength with the electrolyte and optimum connectivity of the constituent phases including the pore phase. Achieving high interface strength often conflicts with the goal of obtaining high porosity. Therefore, we need to complete the cathode processing with minimum shrinkage. This is only possible when we have a mixing state with mixing homogeneity and a controllable mixing scale in the particle packing structure of the composite cathode because the fine scale of the matrix essentially controls the sintering conditions for desirable cathode structures and, consequently, performance.

Like the variation of the optimum composition of composite cathodes discussed above [11,12,15,16], Wilson et al. reported, based on their 3D reconstruction work using high resolution scanning electron microscopy (SEM) and focused ion beam (FIB), that approximately 28 percent of the triple phase boundary identified microstructurally were isolated from the main network, referred to as “inactive [17]”. The initial powder used in their study had particle sizes of 3 and 0.5 μm for LSM and YSZ, respectively, and the composition was LSM/YSZ = 48/52 percent by volume. In this study, commercial LSM (Praxair) and YSZ (Tosoh) powders were used, and this is described as the most common type of electrode. Judging from the particle size difference and composition, the YSZ phase may act as a matrix phase, while the LSM acts as a dispersed phase. Therefore, in order to obtain the maximum triple phase boundary length, it is necessary for the LSM particles to form a continuous network structure with the highest porosity, if possible. In this case, the matrix shrinkage is considered insufficient for the dispersed particles to form a continuous network because some LSM clusters remained isolated from the main LSM network structure. Thus, achieving desirable LSM connectivity requires either increasing the content of LSM powder or reducing the LSM particle size, while keeping the packing density constant. Otherwise, the packing density of the composite cathode should be increased to reduce the interparticle spacing between LSM particles, decreasing the critical matrix shrinkage for LSM clustering presumably at the expense of the porosity. This result supports the argument that it is very difficult to obtain an ideal microstructure with high porosity using commercial powders.

As we discussed previously, the majority of previous studies defined the optimum compositions of the composite cathodes without describing the detailed characteristics of the initial powders and their packing behavior [10,11,12,15,16,17]. In addition, the network formation of the dispersed particles under constrained sintering conditions was never properly studied. Without a full understanding of the mixing state and constrained sintering, it is difficult to analyze the microstructure development of composite cathodes and their relationship to the performance of the cathodes. Care should be taken when selecting the starting powders for fabricating composite cathodes, considering the fact that the mixing state and constrained sintering could make a great difference in both processing and microstructure. Therefore, we developed a composite powder synthesis route based on GNP using the dispersed YSZ particles in the LSM precursor solutions, which is called particle-dispersed GNP (PD-GNP), to produce the composite powders with excellent mixing homogeneity [31]. In particular, the nitrate precursors of lanthanum, strontium, and manganese were mixed in distilled water according to a stoichiometric ratio, with the desired amount of glycine, and pre-synthesized YSZ powder was dispersed in a solution with the assistance of a polymer dispersant, followed by self-combustion to produce the LSM-YSZ composite powder. Since the LSM particles synthesized by GNP are very fine and continuous, the particle size and content of the dispersed YSZ particles were used as the primary processing variables for controlling the composite microstructure. With negligible chemical reactions or solid solubility between the component particles, the dispersed particles distributed discontinuously in the matrix will retard the densification rate of the matrix and generate process flaws, such as large pores, pore clusters, and microcracks. In general, the retardation effect of the dispersed particles on the matrix densification has a tendency to increase as their size and/or content increases [22,23,32,33,34,35]. Therefore, the particle dispersed glycine nitrate process (PD-GNP) has been applied to composite powder synthesis with different particle sizes and varied amount of dispersed YSZ particles. PD-GNP makes it is possible to control the sintering behavior of composite cathodes simply by varying the content or particle size of the dispersed YSZ particles, and consequently to produce a functionally gradient multi-layered composite cathode with different pore structure including porosity and pore size with the help of nearly identical matrix sintering.

**Figure 1:**
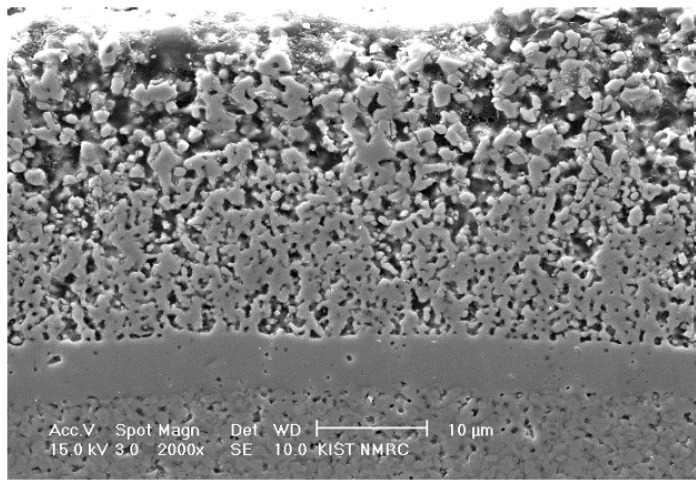
Cross-section image of the cell with functionally graded tri-layer LSM-YSZ composite cathode.

Figure 1 shows a composite cathode structure in a unit cell composed of three functional gradient LSM-YSZ layers that was fabricated by applying three PD-GNP composite powders containing dispersed YSZ particles of different sizes in each. Specifically, the bottom, middle, and top layers contain YSZ particles with average sizes of 0.02 (Nextech materials, Lewis Center, OH, USA), 0.2 (Tosoh Co., Tokyo, Japan), and 2 μm (Unitec Ceramics, Stafford, UK), respectively. The volume ratio of YSZ and LSM was 50:50, and individual layers were sequentially screen printed on top of YSZ electrolyte, followed by sintering at 1100 °C. As expected, the particle size of the dispersed YSZ phase had a significant effect on the microstructure and performance of the composite cathodes. This tri-layered composite cathode was designed to satisfy the strong interface adhesion to the electrolyte and simultaneously to obtain pore structure favorable for efficient gas transport. All of the constituent layers possess PD-GNP LSM-YSZ composite powder of identical composition, in which the LSM matrix in each layer will experience a different level of the constraining effect by the dispersed YSZ particles of different sizes. Attempts were made to obtain the intended microstructure by focusing on the top layer with the largest YSZ particle size forming a continuous YSZ network structure because the bottom layer usually produces a strong interface with the electrolyte layer, even at lower sintering temperatures. The microstructural characteristics of the individual component of graded electrode is summarized in Table 2. The process design for a functionally gradient composite cathode in this study is very effective and powerful because all of the component layers contain almost exactly identical matrix powder.

**Figure 2:**
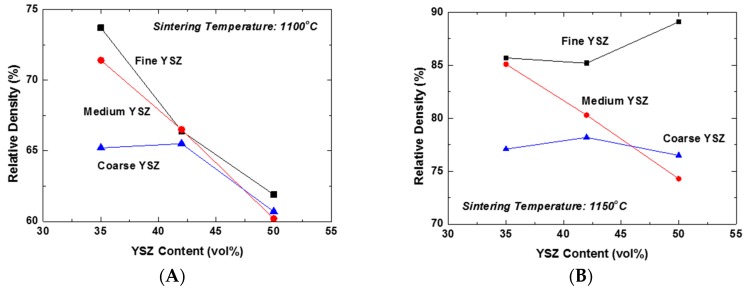
Relative density of LSM-YSZ composites as a function of YSZ content and particle size of YSZ sintered at (**A**) 1100 and (**B**) 1150 °C.

The effect of the content and particle size of YSZ on the constrained sintering behavior of the LSM-YSZ composite was investigated under free sintering conditions. Figure 2A shows the plots of the relative density of LSM-YSZ composites as a function of YSZ content for the YSZ powders of different particle sizes sintered at 1100 °C. The YSZ powders with average sizes of 0.02, 0.2, and 2 μm were designated as fine, medium, and coarse YSZ, respectively. Essentially all of the composites displayed the constraining effect of dispersed YSZ particles on the composite densification at this temperature, except for the samples containing 35 vol % coarse YSZ. This result can be attributed to the extended constraining stress fields around the coarse YSZ particles and their interactions. However, increasing the content of YSZ particles will produce a great number of partial clusters with fine and medium YSZ powders, which might be able to extend the stress field far from them. Accordingly, the constraining effect of YSZ dispersed particles on the matrix densification becomes almost independent of their content at 42 vol % and higher. In contrast, the relative density of the composite increased significantly at 1150 °C compared to 1100 °C, as shown in Figure 2B. There are several interesting aspects to be discussed. The first aspect is that the relative density of the composites containing 42 vol % fine YSZ and higher actually demonstrated an increasing trend, unlike the others. This result indicates that the clusters formed by highly-sinterable fine YSZ particles actually promoted the densification of the matrix and consequently the composites. However, the second aspect to be considered is that the decreasing trend of relative density with YSZ content was persistent for the composites containing the medium YSZ particles. Finally, the relative density of composites containing coarse YSZ particles showed almost negligible variation, indicative of their strictly local constraining effect on the matrix densification. Here, we need to distinguish the global constraining effect from the local one generally accepted in composite sintering. The unexpectedly enhanced densification of the composite containing 50 vol % fine YSZ at 1150 °C is a clear evidence of the exceptional effect of global cluster, i.e., the three-dimensionally continuous network structure, on the highly-sinterable LSM matrix.

**Figure 3:**
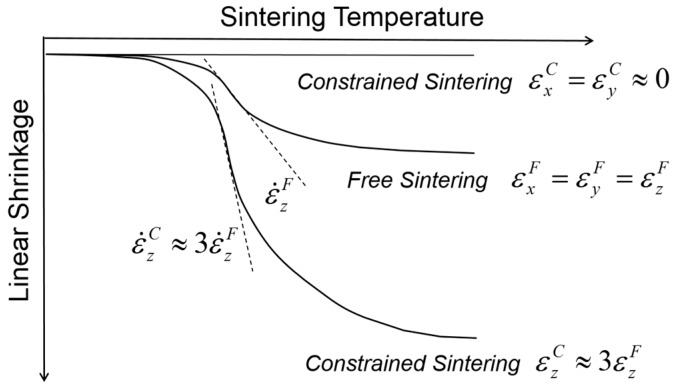
Comparison of shrinkage behavior between the free sintering of bulk alumina and the constrained sintering of alumina thick film on a rigid alumina substrate.

So far, we have discussed the constrained sintering behavior of the composites developed by PD-GNP composite powders containing various YSZ powders of different sizes in free sintering conditions. It was demonstrated that the local constraining stress field around the dispersed YSZ particles can be extended to the global scale as their clusters grow to form a three-dimensionally continuous network structure. However, the presence of a rigid substrate can change the clustering behavior of dispersed particles from three-dimensional to one-dimensional growth due to the uniaxial matrix shrinkage. This phenomenon might be strongly supported by Guillion et al.’s studies in which they actually measured the shrinkage anisotropy in the constrained sintering of alumina thick films on a rigid alumina substrate, as schematically presented in Figure 3 [19,20]. Figure 3 shows a comparison of the shrinkage behavior between the free sintering of bulk alumina and the constrained sintering of thick alumina film on a rigid alumina substrate. As expected, the thick films imposed globally by the external stress field have clear uniaxial shrinkage in the direction perpendicular to the substrate, i.e., the tensile stress field, while the bulk alumina samples in free sintering show isotropic shrinkage in all directions. The shrinkage measured in the *z*-axis was almost three times larger in constrained sintering than in free sintering, satisfying the identical volume shrinkage. In addition, the isothermal sintering study clearly indicates that the shrinkage rate, i.e., the slope of shrinkage curve, also follows the corresponding difference, indicating that the shrinkage rate in the *z*-axis is also approximately three times higher in constrained sintering than in free sintering. It should be noted that the transient stress generated in differential sintering is proportional to the difference of the shrinkage rate between the components involved in the constrained sintering [36]. With essentially no substrate shrinkage, the shrinkage rate of the thick films in the *z*-axis plays a decisive role in the generation of process flaws: for a given packing heterogeneities, such as agglomerates, it is more probable for the thick films in constrained sintering to be subjected to the structural damages or failure under identical sintering conditions than the bulk in free sintering.

**Figure 4:**
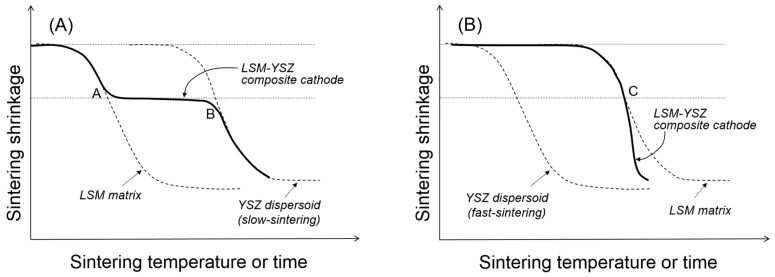
Sintering behavior of LSM-YSZ composite with (**A**) slow- and (**B**) fast-sintering YSZ particles dispersed in LSM matrix under free sintering condition.

If we consider the external constraints imposed by the rigid substrate in the sintering of composite cathodes, it is necessary to first discern the clustering behavior of the dispersed particles in comparison to those in free sintering. To explain the microstructure and properties of the composite cathodes, it is important to understand the clustering of dispersed particles along with uniaxial matrix shrinkage, their cluster orientation, and cluster-cluster interactions in geometric transition. As previously described, the uniaxial matrix shrinkage forces the dispersed particles to form clusters and grow in their size in a preferred orientation. The presence of the dispersed particles will retard the matrix shrinkage considerably due to the local constraints in the matrix around them, and the magnitude of the constraining stress field is expected to increase with the clusters growing in their size [23,34]. Upon forming continuously-connected one-dimensional clusters, the sintering stress of the matrix takes part in the further structural evolution of the clusters. If the sintering stress generated by the matrix is sufficiently large relative to the cluster rigidity, the clusters cannot maintain their structural integrity, and the densification of the composite continues until forming more stable clusters with an increased particle coordination number. In contrast, if the sintering stress by the matrix is not large, relative to the cluster rigidity, the clusters will be structurally persistent throughout the composite sintering. Although the former case has been scarcely reported in composite cathodes, it could be observed in the LSM-YSZ composite cathode containing approximately 50 vol % fine YSZ powder when it was sintered at 1150 °C, as shown in Figure 2B. The competition between cluster rigidity and matrix sintering is summarized in Figure 4. When the YSZ particles disperse in the LSM matrix via slow sintering (Figure 4A), there will be two transition points where the clusters will go through structural transitions. At point A, the dispersed particles will form a three-dimensionally continuous network structure which is considerably resistant to the matrix shrinkage. However, once the sintering temperature is sufficiently high, the clusters of dispersed particles will resume their shrinkage. However, when the YSZ particles dispersed in the LSM matrix via fast sintering (Figure 4B), they are not allowed to form clusters unless the LSM matrix starts to shrink. Thus, the YSZ dispersed particles will form clusters at point C, but those YSZ clusters will enhance the composite sintering.

**Figure 5:**
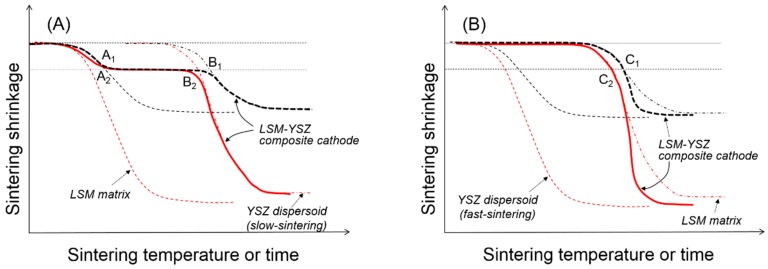
Sintering behavior of LSM-YSZ composite with (**A**) slow- and (**B**) fast-sintering YSZ particles dispersed in LSM matrix under constrained (red) and free (black) sintering conditions.

Compared to the free sintering of bulk composites in Figure 4, Figure 5 shows a schematic presentation of the composite sintering in the presence of the external constraints imposed by the rigid substrate. Assuming that only uniaxial shrinkage is allowed, the linear shrinkage is about three times greater in the presence of the rigid substrate [19], but the critical shrinkage for the clustering of dispersed particles is identical to that in free sintering. When the dispersed particles form critical clusters covering the entire volume of the component are sintered in their respective geometry, the coordination number of dispersed particles in critical clusters will be substantially smaller under the external constraint than under the internal constraint. For the fast-sintering LSM matrix in Figure 5A, the dispersed YSZ particles will form critical clusters connected from the substrate to the top surface at point A_2_ and maintain their integrity until point B_2_ where the clusters are collapsed by the increased matrix sintering stress. For the slow-sintering LSM matrix in Figure 5B, the composite sintering is still controlled by the continuous LSM matrix because the fast-sintering dispersed particles are separated from each other without diffusion paths. However, as the LSM matrix starts shrink, the YSZ dispersed particles begin to interact with each other to form small clusters, finally forming critical clusters at point C_2_. Upon formation of these critical clusters, the fast-sintering YSZ particles control the composite sintering, enhancing the matrix sintering.

Once the dispersed YSZ particles of slow-sintering form critical clusters throughout the LSM matrix at point A_2_ in Figure 5A, all of the component phases, including LSM, YSZ, and pores are continuous, leading to the maximum triple-phase boundary length. If the composite sintering was terminated before point A_2_, there is likely incomplete clustering of dispersed YSZ particles. The 3D analysis work by Barnett et al. revealed that approximately 30% of triple-phase boundary length is isolated from the total network, which might have a composite packing structure consisting of relatively large LSM particles dispersed in YSZ matrix. In this case, the composite sintering should be carried out until the LSM-dispersed particles form critical clusters. Without complete clustering of the dispersed particles, those partial clusters cannot manage to cover the entire cathode space, resulting in the substantial loss of the triple-phase boundary length. If the composite sintering is carried out past point A_2_, there will be well-developed phase connectivity of all constituent phases, which will experience coarsening or grain growth with almost constant porosity. The loss of the triple-phase boundary length through coarsening is expected to be relatively small compared to the partial clustering of dispersed particles.

The slow-sintering dispersed particles can provide a wide operational window by forgiving slight over-firing. However, when the dispersed YSZ particles are fast sintered, it is extremely difficult to identify the transition point C_2_ in Figure 5B for the formation of critical clusters because the composite sintering will be accelerated as soon as fast-sintered YSZ particles form critical clusters. In this case, even though we optimize the sintering conditions of the composite cathode, the microstructure and performance of the composite cathode will suffer from reproducibility. We have, therefore, developed a composite powder synthesis route by the PD-GNP method that provides a composite system with a substantial difference in the sintering rate between the component powders. Simply changing the size and content of dispersed particles in the common matrix at the extremely fine scale allows us to control the sintering behavior and microstructure development without sacrificing the porosity. In addition, the common matrix powder offers us an easy and simple method for process control over the multilayered composite with a hierarchical microstructure for the best structural and electrochemical performances.

## 3. Constrained Sintering of Dense Electrolyte Layer

Unlike the porous composite cathodes, the constrained sintering of a dense electrolyte layer requires the clustering behavior of dispersed particles to be modified in order to allow for continuous densification even after the formation of critical clusters covering the entire electrolyte space. In doing so, the composite shrinkage behavior in Figure 5B might be more favorable for obtaining a dense layer on a rigid substrate. To mimic the clustering of the dispersed particles in the composites, bimodal packing can be employed in the electrolyte of a single phase with the component powders possessing substantially different sintering rates [18,37,38]. This procedure has been successfully applied in the development of an extremely thin bilayer electrolyte by chemical solution deposition route in which YSZ nanoparticles were incorporated in the YSZ precursor chemical solutions [5,8,13,14]. Upon removal of organic substances, the electrolyte layers deposited on a rigid substrate are composed of YSZ nanoparticles dispersed in a continuous YSZ sol particle matrix much finer than the dispersed YSZ nanoparticles.

**Figure 6:**
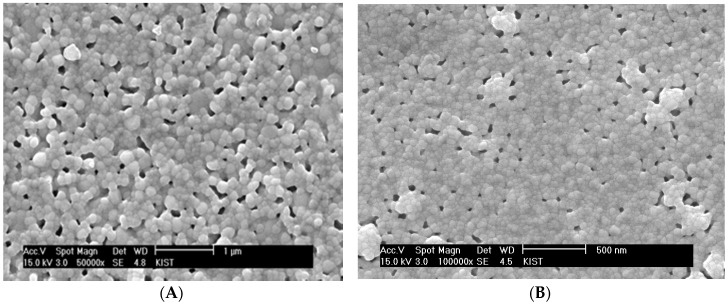
Sintered surface of a YSZ thin electrolyte layer obtained with the chemical solution containing (**A**) 9 and (**B**) 18 vol % YSZ nanoparticles.

Figure 6A shows a SEM micrograph of the sintered surface of YSZ thin electrolyte layer obtained with the chemical solution containing 9 vol % YSZ nanoparticle based on final solid yield. The size of YSZ nano-particles (Nextech, Lewis Center, OH, USA) is 10 nm. Although no significant process flaws were observed, a substantial residual porosity remained at the given sintering temperature of 1100 °C. The sintering temperature needs to be kept as low as possible in order to keep the grain size smaller than the film thickness for its thermodynamic stability. The significant residual porosity can be attributed to extensive cluster rigidity against the matrix shrinkage (Figure 5A). Considering the clustering behavior of the dispersed particles in the presence of the rigid substrate, the film densification appears to be significantly retarded by the clusters covering the whole electrolyte film in three dimensions rather than one dimension. As discussed previously, the dispersed YSZ nanoparticles initially form uniaxial local clusters and then grow to critical clusters covering the film thickness one-dimensionally. Thus, in order to impose the constraining effect on the electrolyte densification, it is necessary for those critical clusters to grow to a global cluster covering the electrolyte volume three-dimensionally. For the electrolyte films prepared by spin coating of chemical solutions containing YSZ nanoparticles, the inter-particle spacing can be estimated based on the principles of the colloidal systems [39]. The interparticle spacing (IPS) is expressed as:

(1) $$IPS=2r\left({\left(\frac{\phi_{m}}{\phi }\right)}^{1/3}-1\right)$$

where *r* is a particle diameter; *φ_m_* is the maximum particle packing fraction; and *φ* is the particle volume fraction. The estimated interparticle spacing in the films containing 9 vol % YSZ nanoparticles is approximately 5 nm, assuming that the particle size is 10 nm and the maximum achievable fractional packing density is 0.3, which is estimated from the powder compact. Extensive local clustering can readily occur in the presence of ever-present microstructural heterogeneities due to the extremely small inter-particle spacing. Even under globally-constraining stress fields exerted by the rigid substrate, there will still be significant interactions between the unidirectional clusters to develop three-dimensionally continuous clusters. Thus, the residual porosity in Figure 6A might be attributed to the three-dimensionally continuous clusters whose rigidity is strong enough to resist matrix sintering. The residual pores in Figure 6A are located mostly around the clusters formed by relatively large YSZ grains, which is consistent with the observation in the sintering of composites containing slow- or non-sintering particles. If the clusters form prematurely in the early stage of sintering, large pores remain around them due to the lack of highly sinterable matrix particles as shown in Figure 6B for 18 vol % YSZ nanoparticles.

**Figure 7:**
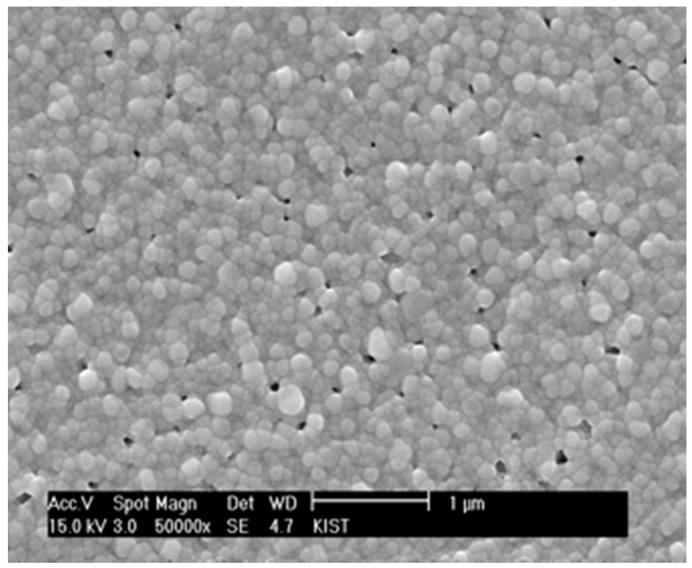
Sintered surface of a YSZ thin electrolyte layer obtained with the chemical solution containing 5 vol % YSZ nanoparticles.

Since the cluster rigidity was too strong for the matrix to continue densification, the content of YSZ nanoparticles incorporated into the chemical solution was reduced to 5 vol % based on YSZ solid yield. Figure 7 shows that the residual porosity was significantly reduced in combination with the reduction of pore size. More importantly, there is almost no evidence for the unidirectional clusters interacting with each other. However, if the content of YSZ nanoparticles is decreased to 2 vol % and lower, the porosity increases, apparently indicating that the interactions between the local constraining stress fields around the nanoparticles are limited, and the matrix is allowed to freely undergo differential sintering in the absence of local constraints. In consideration of the estimates that the inter-particle spacings for the films containing 2 and 5 vol % YSZ nanoparticles are 14.7 and 8.2 nm, respectively, the films prepared by the chemical solution route need to maintain the inter-particle spacing of the slow-sintering nanoparticles below 15 nm and above 5 nm. The optimum content of YSZ nanoparticles in the chemical solution should be determined by the criteria that extensive three-dimensional clustering must be avoided and that relatively unconstrained sintering of the matrix sol particles should be subdued. In our study, the proper inter-particle spacing of the slow-sintering YSZ nanoparticles appears to be approximately 8 nm, which was satisfied because the particle size was 10 nm and the content was 5 vol %.

**Figure 8:**
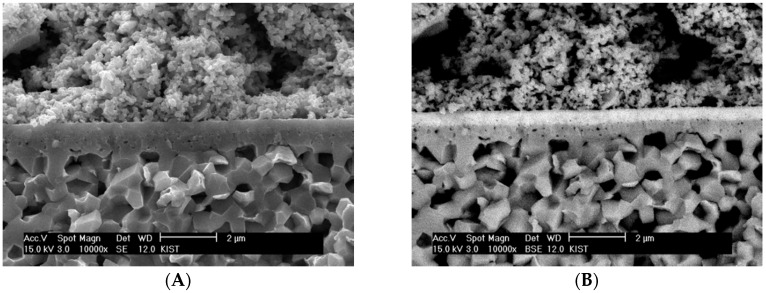
SEM images of the bi-layer electrolyte fabricated by chemical solution deposition route collected in (**A**) secondary electron and (**B**) back-scattered electron modes.

The chemical solution deposition was also applied to the bilayer electrolyte elaboration, as previously reported [14]. Figure 8 shows SEM micrographs of the bilayer electrolyte applied to SOFC cell by chemical solution deposition. In the secondary electron mode in Figure 8A, the component layers constituting the bilayer electrolyte cannot be distinguished, but the GDC layer in white can be clearly distinguished from the YSZ layer in dark in the back-scattered electron mode in Figure 8B. Both of the component layers were fabricated by spin-coating the chemical solutions containing approximately 5 vol % nanoparticles based on solid yield. Despite the global constraints imposed by the rigid substrate, there were no major process flaws in either layer, suggesting that the transient stresses generated during constrained sintering could be reduced to a controllable level by carefully adjusting the size and rigidity of clusters formed by the dispersed particles with their content and size.

## 4. Summary

The post-firing process frequently employed in SOFC fabrication is inevitably confronted with constrained sintering by the plane tensile stress field imposed by the rigid substrate. In addition, the fluctuation of the optimal composition of composite cathodes can be attributed to varied packing structure, including the powder characteristics of the initial powders and their packing structure under the constrained sintering condition. The composite powders prepared by PD-GNP synthesis can provide an alternative solution by hierarchically designing a multilayered composite cathode with dispersed particles of different sizes. Whether the films deposited on the rigid substrate are composite cathodes or bimodal packing electrolytes, the clustering of dispersed particles in the matrix has significant effects on the processing, as well as microstructure, especially in the presence of external constraints imposed by the rigid substrate. Since the film shrinkage takes place preferentially in the direction perpendicular to the substrate, the clusters formed by the dispersed particles tend to be oriented correspondingly. The local constraints developed around the dispersed particles and their clusters are effective for suppressing major process flaws that are otherwise readily generated in the presence of the rigid substrate. The final microstructure should be optimized by adjusting the size and rigidity of the clusters, which can be controlled simply by adjusting the content and particle size of the dispersed particles.

The porous composite cathodes prefer relatively strong rigidity of the clusters covering the entire cathode volume to obtain a stable pore structure, including porosity and pore size favorable for gas transport. However, the dense electrolyte favors limiting the interactions between the uniaxial clusters, which might allow the fine matrix to continue densification by overcoming the cluster rigidity due to the low particle coordination numbers in the clusters. Since the thermodynamic stability of the thin electrolyte layer requires the additional condition that the grain size should be smaller than the film thickness, care should be exercised in selecting the correct size of the dispersed particles employed in the chemical solution deposition route.

## Figures and Tables

**Table 1:** Summary of key processing parameters used by several research groups to determine the optimum composition of GDC/LSCF composite cathodes.

Reference	Powder Characteristics		Consolidation Method	Sintering Condition	GDC/LSCF by Vol %	Polarization Resistance
	GDC	LSCF				
[11]	2.3	0.7	Slurry painting followed by cold isostatic pressing	800–900 °C/2 h	36/64	0.6 Ω∙cm^2^ @590 °C
[15]	2.3	0.7	Spin coating	900 °C/2 h	50/50	0.33 Ω∙cm^2^ @600 °C
[16]	0.16	0.47	Screen printing	1000 °C/2 h	45/55	0.22 Ω∙cm^2^ @700 °C
[12]	Nextech	GNP	Screen printing	975 °C/2 h	65/35	0.27 Ω∙cm^2^ @600 °C

**Table 2:** Structural characteristics of the functionally graded tri-layer LSM-YSZ composite cathode.

	YSZ Particle Size (μm)	Porosity (%)	Average Pore Diameter (μm)	Thickness (μm)
Top Layer	2	43	1.31	7
Middle Layer	0.2	39	1.03	7
Bottom Layer	0.02	36	0.88	6
